# Characterization of Inflammation in In Vivo Mouse Myocardium Using Longitudinal Rotating Frame Relaxation Time (T_1ρ_) and T_2_ Relaxation Time Maps

**DOI:** 10.1002/nbm.70304

**Published:** 2026-04-29

**Authors:** Elias Ylä‐Herttuala, Haja Sherief Nazimutheen Mustafa, Muhammad Arsalan Khan, Mari Merentie, Galina Wirth, Svetlana Laidinen, Lari Holappa, Seppo Ylä‐Herttuala, Timo Liimatainen

**Affiliations:** ^1^ Department of Biotechnology and Molecular Medicine, A.I. Virtanen Institute for Molecular Sciences University of Eastern Finland Kuopio Finland; ^2^ Clinical Radiology Unit, Imaging Center Kuopio University Hospital Kuopio Finland; ^3^ Department of Computer Science, Electrical Engineering and Mathematical Sciences Western Norway University of Applied Sciences Bergen Norway; ^4^ Department of Physiology, Development and Neuroscience University of Cambridge Cambridge UK; ^5^ Heart Hospital Nova, Central Finland Health Care District Jyväskylä Finland; ^6^ Heart Center Kuopio University Hospital Kuopio Finland; ^7^ Research Unit of Health Sciences and Technology University of Oulu Oulu Finland; ^8^ Department of Diagnostic Radiology Oulu University Hospital Oulu Finland

**Keywords:** adenoviral vectors, cardiovascular MRI, inflammation, rotating frame relaxation times

## Abstract

Cardiac magnetic resonance imaging (MRI) is a gold standard to assess functional and anatomical properties of the living heart. Inflammation changes the myocardial tissue and, furthermore, MR relaxation properties. Continuous‐wave (CW) longitudinal rotating frame relaxation time (T_1ρ_) mapping has been used to assess myocardial fibrosis and inflammation. Conventional T_2_ relaxation time is a known marker of edema in the myocardium. In this study, we assessed myocardial inflammation after viral infection in a mouse heart using CW‐T_1ρ_ and T_2_ relaxation times. Adenoviral human vascular endothelial growth factor‐A_165_ (AdVEGF‐A_165_) and empty control adenoviral vector with cytomegalovirus promoter (AdCMV) gene transfers were used to induce inflammation in the mouse myocardium. In vivo CW‐T_1ρ_ and T_2_ relaxation time measurements were performed in both groups (AdVEGF‐A_165_ and AdCMV) after −1‐, 1‐, 3‐, 7‐, 14‐, 21‐, and 28‐day post‐injection. The inflammation associated with gene transfer was verified by hematoxylin and eosin staining after 14‐day post‐injection. One day after AdVEGF‐A_165_ and AdCMV injections and inflammatory reactions, CW‐T_1ρ_ showed a significant increase, which stayed increased as a function of time. T_2_ also increased significantly after both injections and inflammatory reactions as compared to before injections. Contrast difference between inflammation and remote areas was visually observed in both groups in CW‐T_1ρ_ and T_2_ maps. Hematoxylin and eosin staining revealed the area of inflammation after Ad injection in both groups after 14‐day post‐injection. This study showed that both acute and chronic phases of the inflammatory reaction in mouse myocardium caused by myocardial adenoviral injections were associated with increased CW‐T_1ρ_ and T_2_ relaxation time constants. Furthermore, the inflammatory reaction can be followed up with rotating frame and conventional relaxation time mappings.

## Graphical abstract

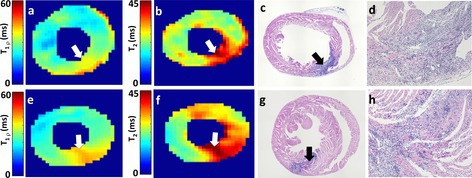

## 1 Introduction

Cardiovascular diseases (CVD) are the leading cause of death worldwide and inflammation is often related to the progression of CVD [1, 2]. It is known that the inflammatory reaction consists of an infiltration of inflammatory cells. Macrophages, monocytes, mast cells, and lymphocytes, among others, secrete a range of cytokines initiating and propagating myocardial interstitial fibrosis activity [3]. Inflammation and fibrosis progression are a general pathway to several myocardial diseases [4].

Cardiac magnetic resonance imaging (MRI) provides accurate functional and anatomical tissue characterization of the myocardium [5, 6]. Edema and inflammation reactions can be determined in myocardium by MRI. Currently, the myocardial pathologies are diagnosed by using late gadolinium enhancement (LGE). Injected gadolinium (Gd)‐based contrast agent accumulates into the extracellular space with slower clearance caused by disrupted microvascular blood circulation as compared to intact myocardium [7]. The LGE reveals extracellular matrix expansion, but the pathology behind the expansion remains unclear. Another downside of Gd‐based imaging is that it cannot be used in patients with renal malfunction [7, 8].

Relaxation time maps (T_1_ and T_2_) are applied for cardiac MRI to diagnose and further characterize pathologies including fibrosis, collagen content, and edema, among other tissue changes, without the need of contrast agents. Native myocardial T_1_ varies with multiple intracellular and extracellular factors, for example, due to fibrosis, giving a good anatomical view of the heart [9]. Typically, T_2_‐weighted MRI is used to quantify myocardial edema [6]. The increase of T_2_ relaxation time also identifies myocardial inflammation and provides inflammation information complementary to LGE [6, 8].

The T_1_ in rotating frame or longitudinal rotating frame relaxation time (T_1ρ_) describes typically relaxation along radio‐frequency (RF) field [10, 11]. Signal decay is characterized by a time constant T_1ρ_ during the RF pulse [10, 11, 12]. The RF power (γB_1_, where γ is gyromagnetic ratio and B_1_ applied RF field) in continuous‐wave (CW) T_1ρ_ is typically varying between 100 and 10,000 Hz to overcome local field gradients [11]. With such RF powers, CW‐T_1ρ_ is selectively sensitive for molecular motions with correlation times close to 1/(γB_1_) [10, 11, 12]. For comparison, T_2_ is nonselective in nature with sensitivity to a wider correlation time range, which is a fundamental difference between T_2_ and T_1ρ_ [12]. Chemical exchange between hydroxyl groups or amine groups and water occurs at the same frequency range as CW T_1ρ_ [11]. Increased free water content due to extracellular space expansion leads to an increased CW T_1ρ_ and T_2_. With γB_1_ close to 500 Hz or over, reasonable contrast between the fibrotic areas and remote myocardium can be achieved in humans [13, 14], in pigs at 3 T [15] and in a mouse at 9.4 T [12, 16, 17, 18, 19, 20]. Due to the sensitivity of T_1ρ_ to fairly long (10^−4^ to 10^−1^ s) correlation times, it has the potential to determine the water–macromolecular interactions involved in inflammatory reaction, where free water content in the tissue is increased, and thus a possibility to determine the progression of fibrosis after the inflammation in myocardium [19].

To study the development of the inflammation process with conventional and rotating frame MRI methods, a mouse model of inflammation needs to be used. The immune reactions associated with adenoviral (Ad) gene therapy vectors are generally known, and the inflammatory effects in the mouse myocardium after intramyocardial gene transfer have been previously reported [21, 22, 23]. Thus, adenoviral human vascular endothelial growth factor‐A_165_ (AdVEGF‐A_165_) and empty control adenoviral vector with cytomegalovirus promoter (AdCMV) gene transfer injections can be used to induce inflammation in the mouse myocardium, because the inflammatory side effects of adenoviral intramyocardial gene transfer injections have been previously well characterized in the mouse heart [22, 23]. Optimal low doses of AdVs produced according to good manufacturing practice protocols cause only little inflammation in myocardium, and AdVs have proven to be safe in preclinical and clinical trials [22]. In general, the transgene injection site in the left ventricle (LV) anterior wall includes mostly inflammatory cells in the acute phase 6 days after the gene transfer and a local fibrotic scar area with a lymphocyte‐intensive inflammatory reaction at the injection site in the LV wall 28 days after the gene transfer [22]. The size of the local scar area is about 15%–20% after AdVEGF‐A165 and AdCMV gene transfers [22].

Thus, the aim of this study was to characterize the progressive inflammatory reaction area with CW‐T_1ρ_ and T_2_ relaxation time mappings in mouse myocardium, where the inflammatory reaction was induced by either gene transfer of AdVEGF‐A_165_ or AdCMV.

## 2 Materials and Methods

Total of 22 female C57BL/6J mice, weighing 23–25 g and with age of 24 weeks, were used for the experiments. In vivo MR included functional cine imaging, T_2_ and CW‐T_1ρ_ relaxation time mappings. Measurements were performed 1 day before and 1, 3, 7, 14, 21, and 28 days after AdVEGF‐A_165_ and AdCMV gene transfers. Animal experiments were done under the license numbers: ESAVI‐ 2011‐03841, ESAVI‐2011‐003264, and ESAVI‐08‐07516 granted by the National Animal Experiment Board in Finland. All animal procedures were performed according to the international ethical guidelines for animal laboratory use.

### 2.1 Viral Constructs and Gene Transfer

Ad constructs of VEGF‐A_165_ and “empty” Ad CMV construct with only CMV promoter without actual transgene were used in the study. Ad constructs were created as described before [23].

Under isoflurane inhalation (induction of 4.5% isoflurane, 450 mL/min air and maintenance of 2.5% isoflurane, 250 mL/min air; Baxter International, Deerfield, IL, USA), echocardiography (ECG) guided intramyocardial gene transfer of AdVEGF‐A_165_ (*n* = 12) and AdCMV (*n* = 10) was performed to the left ventricular anterior wall, as previously described [23]. Shortly, a viral dose of 1 × 10^10^ viral particles diluted with 0.9% NaCl in 10 μL was used in gene transfer. The injections were given with a 30‐G needle in 50‐μL syringe (Hamilton Company, Bonaduz, Switzerland) that was connected to a micromanipulator system (VisualSonics Inc., Ontario, Canada). The needle was inserted through the chest between the ribs into the left ventricular wall. Viral constructs injections were performed in 10 μL volume. Analgesic (carprofen 50 mg/mL, Rimadyl, Pfizer Inc., New York, USA and buprenorphine 0.05–0.1 mg/kg, Temgesic, RB Pharmaceuticals, Slough, England) was given after the procedure.

### 2.2 MRI

All in vivo measurements were performed in a horizontal 9.4‐T MR scanner controlled by an Agilent console (Varian Inc., Palo Alto, CA, USA) using a volume transceiver RF coil (Rapid Biomed GmbH, Germany) with a 35 mm inner diameter. Mice were placed in a prone position such that their hearts were close to the magnet isocenter and at the center of an RF coil. A small animal gating device (Model 1022, Small Animal Instruments Inc., NY, USA) was used for monitoring and triggering ECG signals by the electrode needles placed under the skin of the mouse's front paws and respiration signal by the pressure pillow placed under the mouse. The MRI protocol included functional cine MRI, CW‐T_1ρ_, and T_2_ relaxation time measurements.

Triggered cine MR measurements for cardiac functional performance were performed using gradient echo (GRE) sequence with TR = 4.6 ms, TE = 1.9 ms, flip angle = 15°, number of frames 10–20/cardiac cycle, thickness of slice = 1 mm, field of view = 30 × 30 mm^2^, and data matrix size = 256 × 256 [24]. The number of slices to cover the entire LV was 8–10. Short‐axis slice from mid‐ventricular level was selected based on cine MR images for the CW‐T_1ρ_ and T_2_ relaxation time measurements.

For CW‐T_1ρ_ measurements, an adiabatic half passage pulse [25] with duration of 6 ms and peak power of 1.25 kHz was used to flip magnetization into the xy‐plane followed by a CW pulse with varying duration (0, 18, 36, and 54 ms) and peak power of 1.25 kHz of an adiabatic half passage was applied to flip magnetization back to the z‐axis before readout. For T_2_ measurements, adiabatic double Hahn echo preparation [26, 27] with echo times 0, 7, 14, 21, 28, and 35 ms, pulse duration = 1.5 ms and pulse power = 5 kHz were applied. After the preparations, the readout of CW‐T_1ρ_ and T_2_ measurements was performed using a Turbo fast low angle shot gradient echo sequence readout with TR = 3.5 ms, TE = 1.6 ms, flip angle = 25°, thickness of slice = 1.5 mm, field of view = 30 × 30 mm^2^ and data matrix size = 128 × 128. In both CW‐T_1ρ_ and T_2_ relaxation time measurements, there was a 2 s + respiration cycle delay between different weighting images (CW‐T_1ρ_ or T_2_) to give time for magnetization to fully relax before the next preparation pulse. The imaging slice for CW‐T_1ρ_ and T_2_ measurements was selected from the cine images where the biggest effect of inflammation was based on the movement of the myocardium.

### 2.3 Data and Statistical Analysis

All the relaxation time maps were reconstructed using pixel‐by‐pixel analysis with Aedes (http://aedes.uef.fi) in MATLAB (MathWorks Inc., California, USA) platform. The regions of interest (ROI) were drawn over the injection site and remote myocardium. The ejection fraction (EF) was calculated from the cine MR images as EF = 1 − (end systolic volume/end diastolic volume). The statistical software Graph Pad Prism5 (Graph Pad Inc., California, USA) was used to perform statistical analysis. All the values were given as mean ± standard deviation (SD). Two‐way ANOVA with Bonferroni post hoc correction test was used for the separate comparisons between both groups with CW‐T_1ρ_ and T_2_. One‐way ANOVA was also used for a single‐group comparison.

### 2.4 Tissue Processing and Staining

A total of eight mice (four mice from both AdCMV and AdVEGF‐A_165_ groups) were sacrificed for the histology after MR relaxation time measurements at 14 days after AdVEGF‐A_165_ and AdCMV injections as shown in a previous study [23]. The hearts were perfused with phosphate buffer saline, immersion‐fixed in 4% paraformaldehyde in 7.5% sucrose for 4 h and kept in 15% sucrose overnight. The hearts were embedded in paraffin blocks and transversely cut to 4 μm thick sections used for hematoxylin and eosin (HE) staining. The histological images were taken by a light microscope (Nikon Eclipse, Ni‐E, Tokyo, Japan).

## 3 Results

**FIGURE 1:**
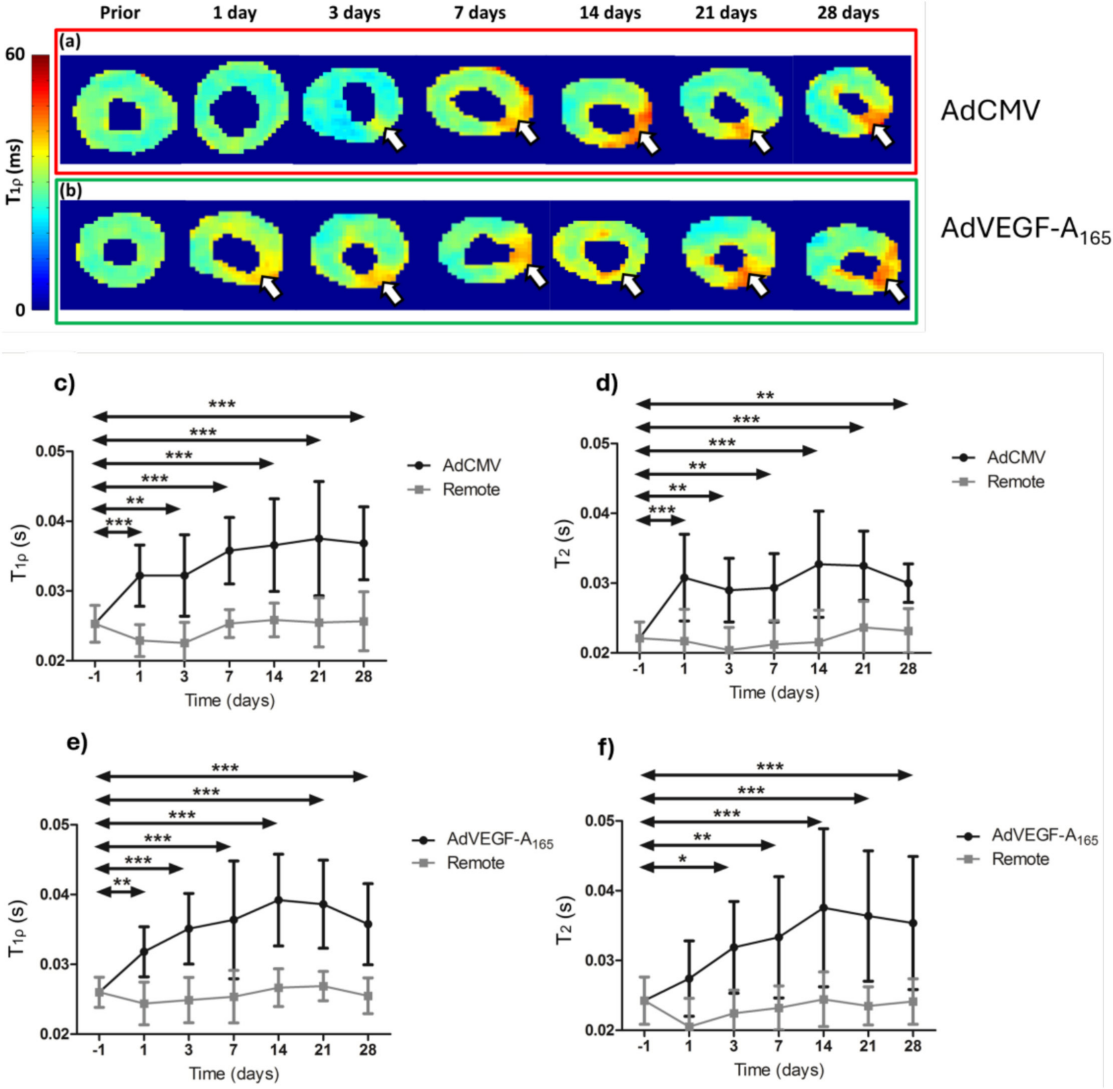
CW‐T_1ρ_ relaxation time maps 1 day prior and 1, 3, 7, 14, 21, and 28 days after AdCMV (a) and AdVEGF‐A_165_ (b), where white arrows indicate the injection sites and the area of inflammation reaction. In (c–f), mean relaxation time constants CW‐T_1ρ_ and T_2_ 1 day prior and 1, 3, 7, 14, 21, and 28 days after AdCMV (c and d, *n* = 10) and AdVEGF‐A_165_ (e and f, *n* = 12) injections from the injection area and remote area (mean ± SD,*p < 0.05, **p < 0.01 and ***p < 0.001 vs. −1 day, two‐way ANOVA, Bonferroni post hoc correction tests).

In vivo CW‐T_1ρ_ and T_2_ relaxation time mappings were performed in both groups along with cine MRI to determine the changes associated with inflammatory reaction in mouse myocardium at different time points after Ad gene transfers. The inflammatory reaction induced by AdVEGF‐A_165_ and AdCMV injections led to a significant increase in CW‐T_1ρ_ and T_2_ relaxation time constants found in both AdVEGF‐A_165_ and AdCMV transduced hearts when comparing before and after the inflammation reaction at different time points (Figure 1). CW‐T_1ρ_ was significantly increased after 1 day of AdVEGF‐A_165_ gene transfer and reached a maximum value of 40.00 **±** 0.01 ms at the 14‐day time point (*p* < 0.001; Figure 1). The maximum value of CW‐T_1ρ_ in AdCMV gene transfer was reached at the time point of 21 days (*p* < 0.001; Figure 1). CW‐T_1ρ_ relaxation time constant stayed increased up to 28 days after AdVEGF‐A_165_ and AdCMV injections (Figure 1). Significant differences were observed with CW‐T_1ρ_ between inflammation reaction area and remote myocardium at all time points after AdVEGF‐A_165_ (*p* < 0.01, *p* < 0.001) and in AdCMV.

T_2_ relaxation time constant showed a significant increase after 3 days of AdVEGF‐A_165_ (*p* < 0.05; Figure 1), whereas a significant increase of T_2_ relaxation time constant was observed at 1 day after the AdCMV (*p* < 0.001; Figure 1). No significant differences in the trends of development of function of time in CW‐T_1ρ_ and T_2_ relaxation time constants at the inflammation area were found between AdVEGF‐A_165_ and AdCMV groups (*p* = 0.51) (Figure 1).

**FIGURE 2:**
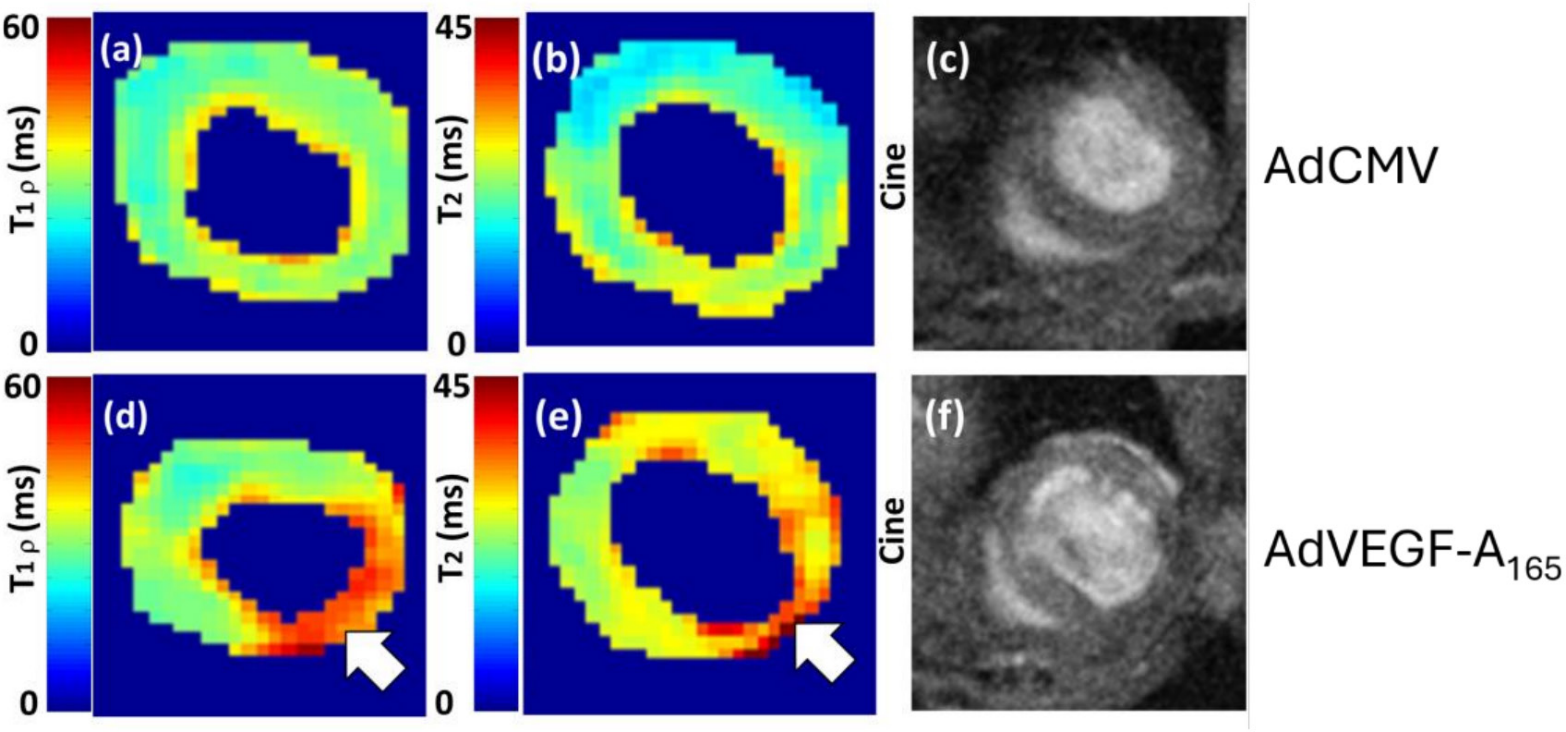
Relaxation time maps after 14 days of AdCMV and AdVEGF‐A_165_ gene transfers. (a) CW‐T_1ρ_ in AdCMV heart. (b) T_2_ in AdCMV heart. (c) Cine MR image of AdCMV heart. (d) CW‐T_1ρ_ in AdVEGF‐A_165_ heart. (e) T_2_ in AdVEGF‐A_165_ heart. (f) Cine MR image of AdVEGF‐A_165_ heart. White arrows indicate injection sites and the area of inflammation reaction.

**FIGURE 3:**
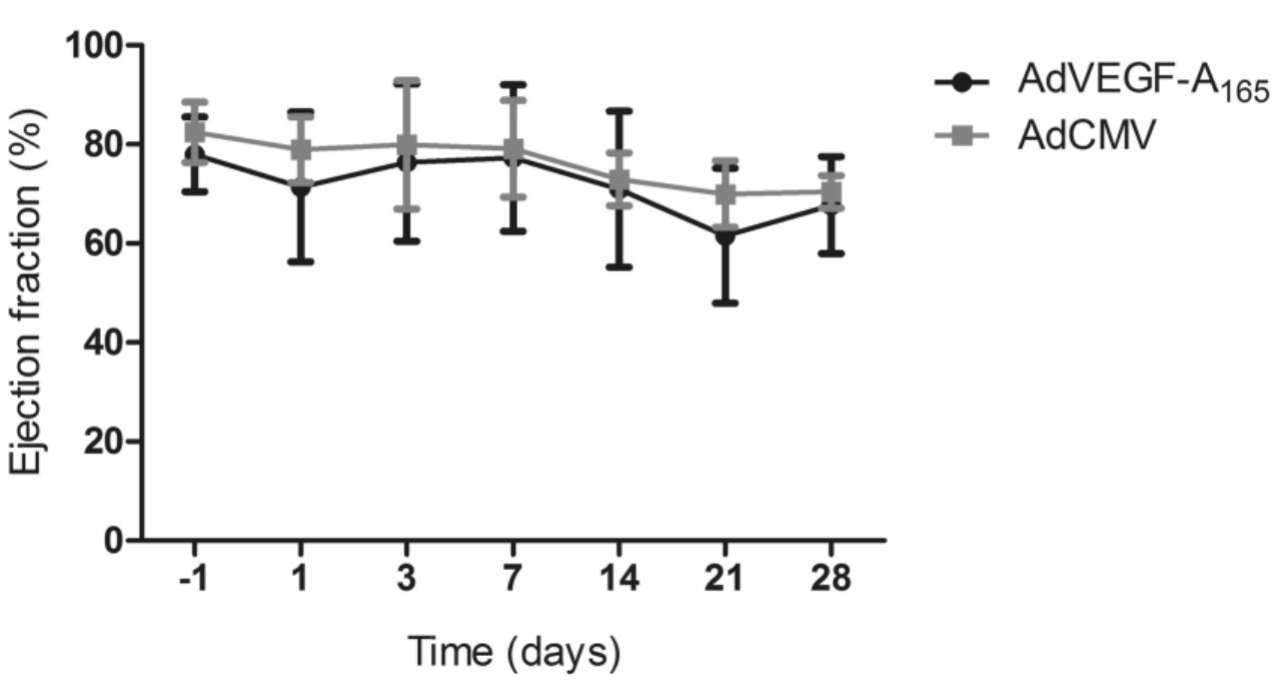
Functional measurements with MRI. Ejection fraction (EF) values (mean ± SD) from AdVEGF‐A_165_ (*n* = 12) and AdCMV mice (*n* = 10).

To observe myocardium at Day 14 after AdCMV and AdVEGF‐A_165_ injections, CW‐T_1ρ_ and T_2_ maps distinguished visually the inflammation reaction area induced with both AdCMV and AdVEGF‐A_165_ from the remote area myocardium (Figure 2). In addition, the cine measurements addressed the cardiac function properties; however, calculated EF did not reveal any significant changes between the time points in both injection groups (Figure 3).

**FIGURE 4:**
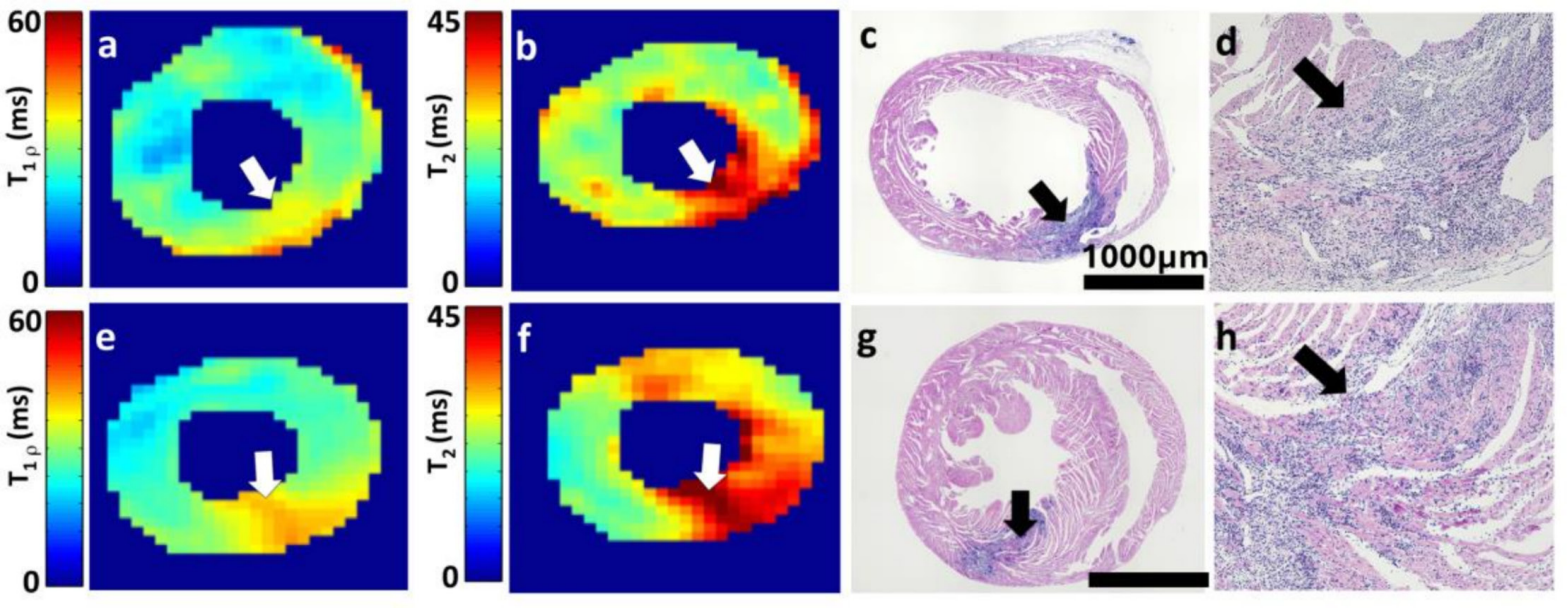
Relaxation time maps and hematoxylin–eosin (HE) stained myocardium after 14‐day post‐injections. (a) CW‐T_1ρ_ map after AdCMV gene transfer. (b) T_2_ map after AdCMV gene transfer. (c) HE stained myocardium after AdCMV gene transfer (10× magnification). (d) HE stained myocardium after AdCMV gene transfer (100× magnification). (e) CW‐T_1ρ_ map after AdVEGF‐A_165_ gene transfer. (f) T_2_ map after AdVEGF‐A_165_ gene transfer. (g) HE stained myocardium after AdVEGF‐A_165_ gene transfer (10× magnification). (h) HE stained myocardium after AdVEGF‐A_165_ gene transfer (100× magnification). White and black arrows indicate inflammation sites.

HE staining revealed scattered infiltration of large amounts of inflammatory cells at the injection site (Figure 4). Additionally, the inflammation injection area had an interstitial fibrosis area and preserved cardiomyocytes in the myocardium (Figure 4). With the best possible co‐registration between MRI and histology, the localization of the inflammation area in the HE staining and in CW‐T_1ρ_ and T_2_ relaxation time maps was similar (Figure 4).

## 4 Discussion

CW‐T_1ρ_ and T_2_ mappings were applied to follow‐up inflammatory reaction after myocardial AdVEGF‐A_165_ and AdCMV viral injections in mice in vivo. This study showed the increase of CW‐T_1ρ_ at the site of injection already early, at 1‐day time point, after injection and staying elevated through the 28 days follow‐up. This was accompanied by both an increase of T_2_ relaxation time constant at the same areas and inflammatory cells and interstitial fibrosis seen at the histological level.

In vivo measurements of CW‐T_1ρ_ showed increased relaxation time constants after 1 day of AdVEGF‐A_165_ and AdCMV injections compared to relaxation time constants before transfer. Increased CW‐T_1ρ_ at the injection site is associated with edema, pure inflammatory reaction, development of necrosis, and interstitial fibrosis in the myocardium, as has been shown previously with this model [22, 23]. Characterizing the actual inflammation reaction areas with CW‐T_1ρ_ in the myocardium, to the best of our knowledge, has not been done before. However, CW‐T_1ρ_ and T_2_ are not sensitive to differentiating the causes of inflammation reaction, in this case, which kind of Adv is causing the inflammation. CW‐T_1ρ_ and T_2_ signal comes from water molecule interactions between its surroundings and, as the inflammation reaction causes an increase of free water, different cell types, and other macromolecules in the area of inflammation, we can determine those interactions with MRI. Authors in the previous article suggested that the adiabatic T_1ρ_ during hyperbolic secant pulses can determine inflammation areas in the mouse myocardial infarction ex vivo model [12, 19, 20, 21] and the myocardial damage after 2 h of myocardial infarct [12]. It is known that adiabatic T_1ρ_ is done differently than CW‐T_1ρ_ [19, 20]. CW‐T_1ρ_ has been used to determine inflammation areas in other anatomies, such as in a liver, where they found that CW‐T_1ρ_ was increased in acute inflammation [28]. Another previous study also showed the increase of CW‐T_1ρ_ in a mouse model after 10 days of transverse aortic constriction [29] and in a mouse model of myocardial infarction [12, 16, 17, 18, 19, 20], where it has been shown that CW‐T_1ρ_ has a potential to be a biomarker for chronic fibrosis characterization. Altogether, the reason for the increase of CW‐T_1ρ_ at the gene transfer site in this study is most likely due to increased free water content in the tissue extracellular space expansion, where chemical exchange between hydroxyl group or amine groups and free water molecules occurs at the same frequency range as RF‐pulse in the CW T_1ρ_ method [11, 28, 30].

It has been previously shown with this mouse intramyocardial gene transfer model that AdCVM and AdVEGF‐A165 injections are associated with inflammation and fibrosis development at the injection site within 28 days, which also led to decreased LV EF after AdVEGF‐A165 gene transfer, but not after empty AdCMV vector injection [22, 23]. In our previous studies, it has been shown that the needle itself or saline injections of similar volume (10 μL) did not cause notable tissue damage nor inflammation when this was evaluated at the 28 days' time point, as only a tiny area of inflammatory cells and scar tissue representing the needle tract was seen in histology [22]. Thus, our MRI results are only from inflammation reaction caused by AdVEGF‐A_165_ and AdCMV viruses. The injection of Ad expressing VEGF‐A_165_ has shown a short‐term acute angiogenic response and the enlargement of capillaries and increased vessel permeability, which were associated with an angiogenic response leading to severe edema and tissue damage [22, 23]. These results support our MRI findings. Increased T_2_ is associated with increased free water content in myocardium, making T_2_ to be a good edema marker for myocardial injuries [18]. The result of this study suggests that T_2_ can be also used to determine inflammation reaction area in mice (Figures 3 and 4); however, exact co‐registration between histology and MRI needs to be done before exactly proving this statement. Because the findings are similar between CW‐T_1ρ_ and T_2_ and between AdVEGF‐A_165_ and AdCMV injections, it can be suggested that CW‐T_1ρ_ relaxation time is also capable to determine inflammation reaction area in myocardium.

HE stainings confirmed inflammation and interstitial fibrosis in myocardium 14 days after gene transfer at the injection sites in LV in both groups, which validates the findings in MRI. The inflammatory reaction in both injection groups is associated with Ad injection, as found in earlier study [23, 24].

A possible partial volume effect near the endocardium needs to be considered because immobile blood near the endocardium in the LV affects relaxation times by increasing them, and thus, these effects might have affected the results. Lastly, the challenge to exactly match the thin histological section and thicker relaxation time maps exists; however, localizing the right ventricular and papillary muscles visually in both MRI and histology section was used to match and verify qualitatively the inflammation site.

## 5 Conclusions

We showed that acute and chronic inflammatory area in mouse myocardium can be associated with increased CW‐T_1ρ_ and T_2_ after intramyocardial adenoviral injection. Inflammation reaction can be followed up with these endogenous relaxation time constant biomarkers.

## Author Contributions

E.Y.‐H., H.S.N.M., and M.A.K. did programming and magnetic resonance imaging, analyzed the data, and wrote the manuscript. M.M. and G.W. contributed to virus injections. M.M., G.W., S.L., and L.H. contributed to surgery, histology, tissue processing, and writing and editing the manuscript. T.L. and S.Y.‐H. contributed to the design, data analysis, writing, and editing of the manuscript. All the authors have read and approved the manuscript.

## Funding

The authors thank Research Council of Finland (340761); Flagship of Advanced Mathematics for Sensing, Imaging and Modelling (359186 and 347445); Sigrid Juselius Foundation; GeneCellNano Flagship; Finnish Cultural Foundation; Finnish Foundation for Cardiovascular Research; State Research Funding for university‐level health research, Kuopio University Hospital, Wellbeing Service County of North Savo; Mauri and Sirkka Wiljasalo Foundation; Paavo Nurmi Foundation; Emil Aaltonen Foundation; Matti and Vappu Maukonen Foundation; Maud Kuistila Memorial Foundation; and Antti ja Tyyne Soininen Foundation for the financial support.

## Conflicts of Interest

The authors declare no conflicts of interest.

## Data Availability

The datasets used and/or analyzed during the current study are available from the corresponding author on reasonable request.
